# Bayesian-Based Hybrid Method for Rapid Optimization of NV Center Sensors

**DOI:** 10.3390/s23063244

**Published:** 2023-03-19

**Authors:** Jiazhao Tian, Ressa S. Said, Fedor Jelezko, Jianming Cai, Liantuan Xiao

**Affiliations:** 1 School of Physics, Taiyuan University of Technology, Taiyuan 030024, China; 2 Institute for Quantum Optics and Center for Integrated Quantum Science and Technology, Ulm University, 89081 Ulm, Germany; 3 School of Physics, International Joint Laboratory on Quantum Sensing and Quantum Metrology, Huazhong University of Science and Technology, Wuhan 430074, China

**Keywords:** optimal control, NV center, quantum sensing, magnetometry

## Abstract

NV centers are among the most promising platforms in the field of quantum sensing. Magnetometry based on NV centers, especially, has achieved concrete development in areas of biomedicine and medical diagnostics. Improving the sensitivity of NV center sensors under wide inhomogeneous broadening and fieldamplitude drift is a crucial issue of continuous concern that relies on the coherent control of NV centers with high average fidelity. Quantum optimal control (QOC) methods provide access to this target; nevertheless, the high time consumption of current methods due to the large number of needful sample points as well as the complexity of the parameter space has hindered their usability. In this paper, we propose the Bayesian estimation phase-modulated (B-PM) method to tackle this problem. In the case of the state transforming of an NV center ensemble, the B-PM method reduced the time consumption by more than 90% compared with the conventional standard Fourier basis (SFB) method while increasing the average fidelity from 0.894 to 0.905. In the AC magnetometry scenario, the optimized control pulse obtained with the B-PM method achieved an eight-fold extension of coherence time $T_{2}$ compared with the rectangular π pulse. Similar application can be made in other sensing situations. As a general algorithm, the B-PM method can be further extended to the open- and closed-loop optimization of complex systems based on a variety of quantum platforms.

## 1. Introduction

The nitrogen-vacancy (NV) center in diamond shows bright prospects in the quantum sensing of magnetic fields [1,2,3], electric fields [4], temperature [5] and strain [6]. In these areas, research on NV center-based ultrasensitive magnetometry has achieved fast development [7,8] and is on the road to practical and commercial applications in biomedicine and diagnostics [9,10,11,12]. Inhomogeneous broadening due to ambient nuclear spins and external bias fields is one of the main obstacles to further improving the sensitivity of sensors. To alleviate the problem, dynamical decoupling (DD) is widely applied in sensing strategies based on NV centers [13,14,15], and various optimal methods are used to increase the performance of DD sequences [16,17,18], since inhomogeneous broadening also damages the fidelity of the control pulses with finite pulse length that construct DD series. Adiabatic strategies can significantly prolong decoherence time $T_{2}$ and sensitivity compared with conventional flat π pulses [17], while smoothly shaped pulse designs based on numerical optimization [18] can adapt to a wider pulse length range where the adiabatic condition is not satisfied. One major drawback of numerical optimal designs is their time efficiency. On one hand, multiple sample points need to be measured to obtain an accurate value of the objective function denoting the average fidelity over frequency and field amplitude broadening, so the processing time for calling the objective function once is prolonged. On the other hand, the total objective function calling times increase along with the number of parameters, while in most cases, a larger number of parameters (over 10) are needed to guarantee fair fidelity. A rapid but well-performed optimization method is anticipated to improve the usability of such control strategies.

In this work, we propose the Bayesian estimation phase-modulated (B-PM) method to overcome the time consumption problem. Unlike the developed Bayesian methods [19,20], the B-PM method grafts the Bayesian estimation model onto the direct search method, thus circumventing the complex process to calculate the acquisition functions [21]. Further taking advantage of the phase-modulated method, the B-PM method makes itself an efficient hybrid optimization method for robust quantum control against noise. The objective function of the optimization process under consideration is the average value of multiple functions with different detuning and amplitude values of the control field. The values of these multiple functions can specifically refer to the fidelity between the final and target states, gate operators and so on. Using the Bayesian-based estimation model, we make an accurate prediction of the average fidelity based on a small number of sample points, so the computation time for calling the objective function once is reduced. In addition, adopting a phase-modulated basisallows the control field to comprise multiple frequency components with fewer parameters, which leads to a significant decrease in the necessary total number of objective function calling times to find the local optimal results. We further verify that the computation time of the estimation process is negligible compared with the sample measuring process. Overall, the B-PM method decreases the total time consumed during the entire optimization process. We firstly applied the B-PM method to the state flipping of an NV center ensemble. Compared with the conventional standard Fourier basis(SFB) method without estimation, the B-PM method increased the average fidelity from 0.894 to 0.905 with only 9.3% of total time consumption. When applied to the sensing strategy of AC magnetic signals, the B-PM-shaped pulse prolonged decoherence time $T_{2}$ by 8 times compared with the conventional rectangular π pulse. The B-PM method could be extended to DC sensing strategies [22], and open- and closed-loop optimization processes for open systems [23,24] and many-body systems [25,26,27].

## 2. Methods

### 2.1. Optimal Control Model of NV Center Ensemble

**Figure 1:**
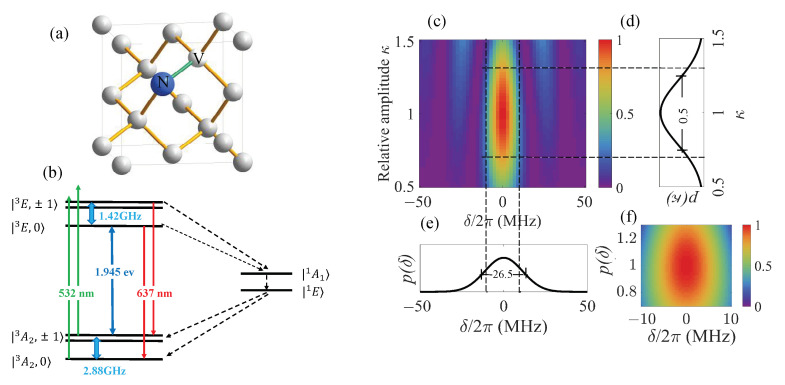
(**a**) Schematic of NV center in diamond lattice. (**b**) Schematic diagram of energy level of NV center. (**c**) Fidelity of state flip of NV center using rectangular control field g(t)=10 MHz. (**d**) Probability density of frequency detuning δ. (**e**) Probability density of amplitude drift factor δ. (**f**) Sampling range of numerical simulation when computing the value of average fidelity F.

The nitrogen-vacancy center (NV center) in diamond [28] has a triplet ground state with electron spin S=1 and zero-field splitting D=2π×2.88 GHz. The energy gap between the ground and excited states is 1.945 ev (637 nm), and due to uneven radiation probability from excited states $|m_{s}=\pm 1\rangle$ and $|m_{s}=0\rangle$ to the radiationless metastable intermediate state, the NV center can be optically initialized and read out. The structure of the NV center and energy level scheme are shown in Figure 1a,b. The Hamiltonian of the triplet ground state of the NV center can be expressed as

(1) $$H=D\left(S_{Z}^{2}-\frac{2}{3}\right)+\gamma B\cdot S+H_{\mathrm{ele}}+H_{\mathrm{hf}},$$

where *D* is the zero-field splitting, B is the magnetic vector, $\gamma_{\mathrm{NV}}=2\pi \times 2.8$ MHz/G is the gyromagnetic ratio of the NV center, $H_{\mathrm{ele}}$ is the electric interaction term with coupling coefficient ∼ Hz/(V/m) and $H_{\mathrm{hf}}$ is the hyperfine coupling term expressing the interactions between the NV center and ambient nuclear spins. Under the magnetic field along the *z*-direction, $B=B_{z}$, and by neglecting the electric coupling term, the Hamiltonian of the two-level subspace formed by $m_{s}=0$ ground state $|\psi_{0}\rangle$ and $m_{s}=-1$ (or $m_{s}=1$) ground state $|\psi_{1}\rangle$ can be written as

(2) $$H=\frac{D+\gamma B_{z}}{2}\sigma_{z}.$$

One simple but requisite control target is to flip all spins in the NV ensemble from one state to another with high average fidelity. Let us consider an ensemble of the two-level system described by Equation (2), which is controlled by a time-dependent field g(t); the Hamiltonian of each spin in the ensemble can be represented as

(3) $$H\left(t\right)=\frac{\omega_{0}+\delta }{2}\sigma_{z}+\kappa g\left(t\right)\sigma_{x},$$

where $\omega_{0}=D+\gamma B_{z}$ is the unperturbed energy gap between $|\psi_{0}\rangle$ and $|\psi_{1}\rangle$, δ is the normally distributed detuning term as a result of inhomogeneous local ambient among the ensemble and κ is the amplitude drift factor varying with different control trials. The probability density of δ and κ can be noted as

(4) $$p\left(\delta \right)=\frac{1}{\sqrt{2\pi }\sigma_{\delta }}e^{-\frac{\delta^{2}}{2\sigma_{\delta }^{2}}}$$

and

(5) $$p\left(\kappa \right)=\frac{1}{\sqrt{2\pi }\sigma_{\kappa }}e^{-\frac{\kappa^{2}}{2\sigma_{\kappa }^{2}}}$$

respectively, where we take the full width at half maximum (FWHM) of p(δ) as 2π×26.5 MHz, corresponding to a dephasing time of $T_{2}^{*}\approx 20$ ns [17], and the FWHM of p(κ) as 0.5. Figure 1c–e depict fidelity

(6) $$f\left(\delta ,\kappa \right)={\left|\langle \psi_{g}|\psi \left(\delta ,\kappa ,T\right)\rangle \right|}^{2}$$

as a function of detuning δ and amplitude drift κ of rectangular control field g(t)=π/T=2π×10 MHz, where *T* is the evolution time, ψ(δ,κ,T) is the final state, and the initial state and the target state are taken as $\psi \left(\delta ,\kappa ,0\right)=\psi_{0}$ and $\psi_{g}=\psi_{1}$, respectively. Based on Figure 1c, we took the sample ranges as δ∈2π×[−10,10] MHz and κ∈[0.5,1.5], as is shown in Figure 1f, and the optimization target was to improve the average fidelity in these ranges.

Taking into account inhomogeneous detuning as well as amplitude drift, the explicit form of the average fidelity can be represented as

(7) F=∫∫dδdκp(δ)p(κ)f(δ,κ).

For fixed δ,κ and *T*, ψ(δ,κ,T) is the function of control field g(t); therefore, it is the function of parameters λ that construct g(t). For example, g(t) can be constructed based on the standard Fourier basis as

(8) $$g_{\mathrm{SFB}}\left(t\right)=\sum_{j=1}^{N_{D}}a_{j}\cos\left(\omega_{j}t+\phi_{j}\right)\cos\left(\omega_{0}t\right)$$

where λ={a,ω,ϕ}, or on the phase-modulated Fourier basis [29] as

(9) $$g_{\mathrm{PM}}\left(t\right)=\sum_{j=1}^{N_{D}}a_{j}\cos\left[\omega_{0}t+\frac{b_{j}}{\nu_{j}}\sin\left(\nu_{j}t\right)\right]$$

where λ={a,b,ν}. The optimization target, therefore, is to find parameters λ that give the highest value of F, while the maximum amplitude of g(t) is limited to $g_{\max}$. We concisely describe this optimization model as

(10) $$\begin{array}{cc} \min & 1-F\\ \;s.t.\; & g\left(\lambda \right)-g_{\max}\leq 0. \end{array}$$

Plenty of optimization methods can be used to solve this problem, with λ being the optimization parameters and F being the objective function. Ideally, the value of F should be calculated by averaging the fidelities of a large number of samples taken from the whole ensemble with several repeated control processes. In practice, this time-consuming process is usually replaced by taking the weighted average of several equidistant samples in a certain range, which can be represented as

(11) $$F_{\mathrm{obj}\;}=N\sum_{k=1}^{M}\sum_{j=1}^{N}p\left(\delta_{k}\right)p\left(\kappa_{j}\right)f\left(\delta_{k},\kappa_{j}\right)$$

where the distribution of δ and κ is assumed to be known; *M* and *N* are the numbers of different values of δ and κ, respectively; and $N={\left[\sum_{k=1}^{M}\sum_{j=1}^{N}p\left(\delta_{k}\right)p\left(\kappa_{j}\right)\right]}^{-1}$ is the normalization constant. Even so, this process still consumes a substantial amount of time. Below, we will show how the Bayesian-based estimation method can be used in the optimization process and combined with the PM method to efficiently reduce the total execution time.

### 2.2. The Estimation Model

One essential task of the optimal process is to build a computationally cheap estimation model, also known as metamodel [30,31,32], of the expensive function. Bayesian statics and analysis methods provide powerful tools to complete this task. From the viewpoint of Bayesian statistics, any event can be endowed with a probabilistic property, whether or not it is a stochastic event. So, undetermined parameter θ in a static model can be taken as a realization of stochastic process θ subject to a prior distribution p(θ), which represents the available knowledge or simple beliefs about θ before any observation of the samples. This prior knowledge needs to be updated with the information in observed sample data s to give the final description of θ, represented by posterior distribution p(θ|s). This relationship is explicitly formulated by the Bayesian theorem as follows:

(12) $$p\left(\theta |s\right)=\frac{L(\theta |s)p(\theta )}{p(s)},$$

where likelihood function L(θ|s)=p(s|θ) is defined as the conditional probability distribution of the given parameters of the data [33].

We limit the considered target true functions to be deterministic, which means that all trials with the same input parameters give identical function values. Following the principle of Bayesian statistics, the deterministic response function y(x) of a *k*-dimension variable $x={\left\{x_{1},x_{2},\ldots ,x_{k}\right\}}^{\prime }$ can be treated as a realization of stochastic process Y(x) [34].

(13) $$Y\left(x\right)=\sum_{h}\beta_{h}f_{h}\left(x\right)+\epsilon \left(x\right),$$

where $f_{h}\left(x\right)$ is a function of x; $\beta_{h}$ are unknown coefficients to be estimated; and ϵ(x) is the error term, which is a random process with zero mean and covariance

(14) $$\mathrm{Cov}\left[x_{i},x_{j}\right]=\sigma^{2}\mathrm{Corr}\left[x_{i},x_{j}\right],$$

where $x_{i}$ and $x_{j}$ are two sets of variables, σ is the process variance and $\mathrm{Corr}\left[x_{i},x_{j}\right]$ represents the correlation. Equation (13) can be regarded as the Bayesian prior of the true response function, in which the right-hand side resembles the form of the linear regression model, except that the errors of different x are correlated rather than independent from each other. We consider the common and simple case where the stochastic process is stationary, and the correlation takes the specific form of [34]

(15) $$\mathrm{Corr}\left[x_{i},x_{j}\right]=\exp\left[-d\left(x_{i},x_{j}\right)\right],$$

where

(16) $$d\left(x_{i},x_{j}\right)=\sum_{h=1}^{k}\alpha_{h}{\left|x_{i,h}-x_{j,h}\right|}^{p_{h}}\;\left(\alpha_{h}\geq 0,p_{h}\in \left[1,2\right]\right),$$

where $\alpha_{h}$ and $p_{h}$ are parameters that need to be determined. This specific form corresponds to a product of the Ornstein–Uhlenbeck process at $p_{h}=1$, which is widely used in physical science models. Furthermore, with the correlation form of Equations (15) and (16), regression parts $\sum_{h}\beta_{h}f_{h}\left(x\right)$ in the stochastic process model can be simply replaced with a constant, μ, without undermining the predictive performance [35]. Hereafter, we use the following simplified stochastic process model:

(17) Y(x)=μ+ϵ(x).

A typical method for analyzing such stationary Gaussian process model is the Kriging method [36], which is flexible and robust for global estimation [37] and is widely applied in fields of spatial statistics [36,38], the design of computer experiments [34] and Bayesian optimization [35]. Besides the ordinary Kriging method, various analysis methods based on the Kriging method as well as the Bayesian approach are also well studied for Gaussian processes with non-stationary correlation and other kinds of stochastic processes [37,39,40,41,42]. Here, we adopt the best linear unbiased predictor (BLUP) of Equation (17) obtained with the Kriging method to form the estimation model. Supposing that the response function has been evaluated at *n* samples $s=\{s_{1};s_{2};\ldots ;\ldots s_{n}\}$ and that the corresponding function values are $y\left(s\right)={\left\{y\left(s_{1}\right),y\left(s_{2}\right),\ldots y\left(s_{n}\right)\right\}}^{\prime }$, the BLUP of y(x) can be represented as [35]

(18) $$\hat{y}\left(x\right)=\hat{\mu }+r^{\prime }\left(x\right)R^{-1}\left(y\left(s\right)-1\hat{\mu }\right),$$

where R is an n×n correlation matrix with entries $R_{i,j}=\mathrm{Corr}\left[s_{i},s_{j}\right]$; $r\left(x\right)=[R\left(s_{1},x\right),\ldots ,$$R\left(s_{n},x\right){]}^{\prime }$ is the n×1 vector of correlation between the errors of the sample and untried input x, with $R\left(s_{1},x\right)=\mathrm{Corr}\left[s_{1},x\right]$; $\hat{\mu }={\left(1^{\prime }R^{-1}1\right)}^{-1}1^{\prime }R^{-1}y\left(s\right)$ is the generalized least-squares estimation of μ, and 1 is the n×1 vector with all entries equal to 1.

By properly selecting the parameters in Equation (16), we can expect a predict function that describes true function y(x) well by only using the limited number of sample points s. One useful method to determine these parameters is maximum likelihood estimation (MLE). According to the Gaussian process we adopt here, the likelihood function of the samples is [35]

(19) $$\frac{1}{{\left(2\pi \right)}^{n/2}{\left(\sigma^{2}\right)}^{n/2}{\left|R\right|}^{\frac{1}{2}}}\exp\left[-\frac{{\left(y\left(s\right)-1\mu \right)}^{\prime }R^{-1}\left(y\left(s\right)-1\mu \right)}{2\sigma^{2}}\right],$$

where |R| represents the determinant of R. The analytical solutions of μ and σ maximizing Equation (19) are

(20) $$\hat{\mu }={\left(1^{\prime }R^{-1}1\right)}^{-1}1^{\prime }R^{-1}y\left(s\right),$$

which is exactly the generalized least-squares estimation of μ, and

(21) $${\hat{\sigma }}^{2}=\frac{{\left(y\left(s\right)-1\hat{\mu }\right)}^{\prime }R^{-1}\left(y\left(s\right)-1\hat{\mu }\right)}{n}.$$

Therefore, we only need to find the values of $\alpha_{h}$ and $p_{h}$ that give the maximum value of Equation (19), which can be completed using any optimization method at hand.

Corresponding to the specific model of the two-level NV electron spin system, once control field g(t) and control time *T* are fixed, target function f(δ,κ) is the deterministic response function of two-dimension variable $x={\left\{\delta ,\kappa \right\}}^{\prime }$ and can be approximately estimated using predict function $\hat{f}\left(x\right)$, which takes the same form of $\hat{y}\left(x\right)$ in Equation (18). Specifically, we write the Hamiltonian in the interaction picture with respect to $\frac{\omega_{0}}{2}\sigma_{z}$ as

(22) $$H_{\delta ,\kappa }\left(t\right)=\frac{\delta }{2}\sigma_{z}+\kappa H_{c}\left(t\right),$$

where $H_{c}\left(t\right)$ is the time-depended Hamiltonian. By neglecting counter-rotating terms in rotating-wave approximation, $H_{c}\left(t\right)$ can be expressed as

(23) $$H_{c}\left(t\right)=\Omega_{x}\left(t\right)\sigma_{x}+\Omega_{y}\left(t\right)\sigma_{y},$$

where $\Omega_{x}\left(t\right)$ and $\Omega_{y}\left(t\right)$ take the forms of

(24) $$\Omega_{x}^{\mathrm{SFB}}\left(t\right)=\sum_{j=1}^{N_{D}}\frac{a_{j}}{2}\cos\left(\omega_{j}t+\phi_{j}\right)\cos\left(\varphi_{j}\right),$$

(25) $$\Omega_{y}^{\mathrm{SFB}}\left(t\right)=\sum_{j=1}^{N_{D}}\frac{a_{j}}{2}\cos\left(\omega_{j}t+\phi_{j}\right)\sin\left(\varphi_{j}\right)$$

with the SFB method and the forms of

(26) $$\Omega_{x}^{\mathrm{PM}}\left(t\right)=\sum_{j=1}^{N_{D}}\frac{a_{j}}{2}\cos\left[\frac{b_{j}}{v_{j}}\sin\left(v_{j}t\right)\right],$$

(27) $$\Omega_{y}^{\mathrm{PM}}\left(t\right)=\sum_{j=1}^{N_{D}}\frac{a_{j}}{2}\sin\left[\frac{b_{j}}{v_{j}}\sin\left(v_{j}t\right)\right]$$

with the PM method. The target response function can be expressed as

(28) $$f\left(\delta ,\kappa \right)={\left|\langle \psi_{g}|U_{\delta ,\kappa }\psi \left(0\right)\rangle \right|}^{2},$$

where

(29) $$U_{\delta ,\kappa }=T\exp\left[-i\int_{0}^{T}H\left(\delta ,\kappa ,t\right)dt\right]$$

is the evolution operator and T is the time-order operator.

So far, we established the Bayesian-based estimation model of the final-state fidelity under certain frequency detuning δ and amplitude bias ratio κ. With this usable tool, we could further construct a high-efficiency optimization method to explore the optimal control field of an ensemble system.

### 2.3. The Hybrid Optimization Method

**Figure 2:**
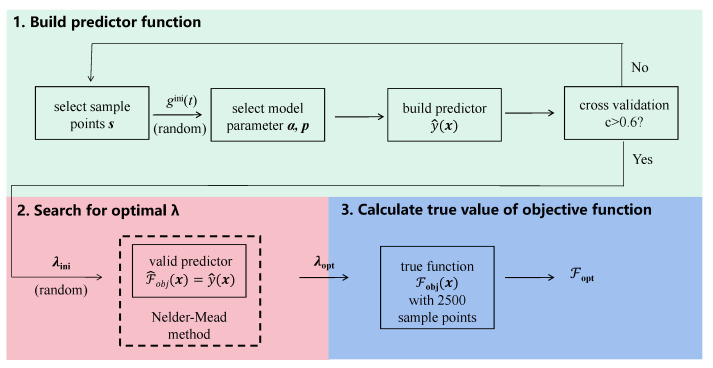
Process schematic of Bayesian-based optimization method.

Now, we introduce the hybrid B-PM optimization method for solving the problem described by Equation (10). As represented in Figure 2, the whole optimization process can be divided into three parts: initial construction of the available predict function; then, search for optimal parameter λ using the predict function as the objective function; lastly, calculation of the true value of the objective function in Equation (11) with M×N=2500 sample points. The first segment is the essential part of this method, and the following strategies are adopted to ensure convergence and improve the optimization results.

1. Sample position: In the sample selection step, instead of completely randomly picking the sample points, we add random bias to evenly distributed coordinate values to obtain the randomly yet uniformly distributed sample positions. This choice improves the estimation performance, especially in cases with small sample numbers.
2. Model parameter: Model parameters α and *p* are selected based on a random initial field $g^{\mathrm{ini}}\left(t\right)$ and are fixed during one optimization process. This tactic ensures the monodromy of the objective function and the convergence of the subsequent search process, reduces the computation time spent building the predict function and does not damage the estimation accuracy.
3. Cross validation: After constructing a predict function, cross validation is applied to eliminate the low-performing ones. Each function value of the *n* sample points y(s) is successively regarded as an unknown quantity and is predicted based on model parameters α and *p*, and other n−1 samples, given a corresponding predict vector $y_{\mathrm{pred}}\left(s\right)$. Then, linear fitting is performed on those points located at $\left(y\left(s\right),y_{\mathrm{pred}}\left(s\right)\right)$, and we use slope $p_{\mathrm{fit}}$ as a criterion. In principle, $p_{\mathrm{fit}}$ far away from 1 indicates bad performance of the predictor, and by examining the fitted slope of bad predict models occurred in the optimization process, we found that bad models correspond to small values of $p_{\mathrm{fit}}$. Therefore, we set $p_{\mathrm{fit}}>0.6$ as the threshold. If this condition is fulfilled, the predict function can be used as the objective function in the following direct search process. Otherwise, a new model needs to be built from the very beginning.

After the valid predictor was constructed, it was used as the objective function of the following direct search process, where we used the phase-modulated method to further improve the efficiency. Initial parameter $\lambda_{\mathrm{ini}}$ was taken in the range of $a_{j}^{\mathrm{ini}}\in \left[0,\Omega_{\max}\right]$, and $b_{j}^{\mathrm{ini}},\nu_{j}^{\mathrm{ini}}\in \left[0,2\pi /T\right]$, where $\Omega_{\max}$ is the maximum field amplitude and *T* is the evolution time. The SFB method was also investigated for comparison, with initial parameters $a_{j}^{\mathrm{ini}}\in \left[0,\Omega_{\max}\right]$, $\omega_{j}^{\mathrm{ini}}\in \left[0,2\pi /T\right]$ and $\phi_{j}^{\mathrm{ini}},\varphi_{j}^{\mathrm{ini}}\in \left[0,2\pi \right]$. In both methods, we took $\Omega_{\max}=2\pi \times 10$ MHz and T=100 ns, and the total field amplitude was constrained as $\sqrt{\Omega_{x}^{2}\left(t\right)+\Omega_{y}^{2}\left(t\right)}\leq \Omega_{\max}$; frequency parameters $b_{j},\nu_{j}$ and $\omega_{j}$ were constrained in range [0,5×2π/T]; and phase parameters $\phi_{j}$ and $\varphi_{j}$ were constrained in range [0,2π]. An extra constraint on $b_{j}/\nu_{j}$ can be added to avoid an infinite value when $\nu_{j}$ goes close to zero, e.g., $b_{j}/\nu_{j}\geq 10^{10}$, but in our simulation, the constraint on $b_{j}/\nu_{j}$ was not used, and no infinite value of $b_{j}/\nu_{j}$ appeared during the optimization process.

In the last step, given optimization parameter $\lambda_{\mathrm{opt}}$, the average fidelity in Equation (11) was calculated based on 2500 uniformly spaced sample points in regions δ∈2π×[−10,10] MHz and κ∈[0.5,1.5], with M=N=50.

### 2.4. AC Magnetometry

Magnetometry is one of the most promising fields in which the NV center can play an important part, with its outstanding characteristics such as fine biological compatibility, normal pressure and temperature operating conditions, and relatively long spin lifetimes. Pulsed and continuous dynamical decoupling (DD) has been applied in the coherent control and magnetic sensing of NV centers. In practice, the large inhomogeneous broadening of an NV center ensemble decreases the controllability of the DD sequence and ultimately hinders the sensitivity in magnetometry. Adiabatic strategies have been applied to tackle such problem and have achieved good results. When the frequency of the signal is not low enough to fulfill the adiabatic condition, finding the optimal field with limited pulse length to achieve certain gate operation becomes a key approach to improve the sensitivity. Here, we used the B-PM method to optimize the control field and obtain robust Pauli-X and Pauli-Y gates under inhomogeneous broadening, which composes the XY-8 DD sequence in the AC magnetometry process.

During the pulse period, control Hamiltonian $H_{c}$ in Equation (22) takes the form of

(30) $$H_{\mathrm{PM}}^{X}\left(t\right)=\Omega_{1}\left(t\right)\sigma_{x}+\Omega_{2}\left(t\right)\sigma_{y}$$

for the Pauli-X gate and

(31) $$H_{\mathrm{PM}}^{Y}\left(t\right)=\Omega_{1}\left(t\right)\sigma_{y}-\Omega_{2}\left(t\right)\sigma_{x}$$

for the Pauli-Y gate, where

(32) $$\Omega_{1}\left(t\right)=\sum_{j=1}^{N_{D}}\frac{a_{j}}{2}\cos\left[\frac{b_{j}}{v_{j}}\sin\left(v_{j}t\right)\right],\;\Omega_{2}\left(t\right)=\sum_{j=1}^{N_{D}}\frac{a_{j}}{2}\sin\left[\frac{b_{j}}{v_{j}}\sin\left(v_{j}t\right)\right].$$

For gate optimization, the objective function takes the form of

(33) $$F_{\mathrm{obj}}=N\sum_{k=1}^{M}\sum_{j=1}^{N}p\left(\delta_{k}\right)p\left(\kappa_{j}\right)f_{g}\left(\delta_{k},\kappa_{j}\right),$$

where

(34) $$f_{g}\left(\delta ,\kappa \right)=\frac{1}{2}+\frac{1}{3}\sum_{\epsilon =x,y,z}\mathrm{Tr}\left(U_{\mathrm{Tar}}\frac{\sigma_{\epsilon }}{2}U_{\mathrm{Tar}}^{†}U_{\delta ,\kappa }\frac{\sigma_{\epsilon }}{2}U_{\delta ,\kappa }^{†}\right),$$

is the gate fidelity of fixed δ and κ; $U_{\mathrm{Tar}}$ is the target quantum gate; $U_{\delta ,\kappa }$ is the actual operator, which takes the same form as in Equation (29); and $N,p\left(\delta_{k}\right)$ and $p\left(\kappa_{k}\right)$ take the same forms and values as in Equation (11). We applied the B-PM method to search for the robust control field with the amplitude constraint of $\sqrt{\Omega_{1}^{2}\left(t\right)+\Omega_{2}^{2}\left(t\right)}⩽\Omega_{\max}$ and obtained an optimized shaped pulse, which constructs the XY-8 DD sequence in the AC magnetometry strategy.

The AC magnetic signal to be measured oscillates at frequency $\omega_{s}$, and the control pulses are applied on time nodes where the AC signal changes its direction. Due to the power limitation of the microwave field, each pulse possesses a finite pulse length $T_{\mathrm{pulse}}$, whose value follows the relationship $\omega_{s}=\pi /\left(T_{\mathrm{pulse}\;}+\tau_{\mathrm{pulse}}\right)$, where $\tau_{\mathrm{pulse}}$ is the time separation between two adjacent pulses. The amplitude of AC magnetic signal $g_{\mathrm{ac}}$ can be read out from the Ramsey oscillation of the NV center sensor. At the beginning of the measurement, the NV centers in the ensemble are initialized into state |0〉 and flipped with a π/2 pulse, and begin to rotate around the $\sigma_{z}$-axis. After a time period *t*, one 3π/2 pulse is applied to the sensors, and their population on |0〉 is measured. Under the ideal condition with instantaneous gate pulses, the population can be expressed as $P_{0}=\left(1+\cos(2\chi (t))\xi (t)\right)/2$, with phase term $\chi \left(t\right)\equiv \int_{0}^{t}g\left|\cos\left(\omega_{s}t^{\prime }\right)\right|dt^{\prime }$ and exponential decay term ξ(t) being induced by inhomogeneous broadening and dynamic noise. At coherent time $t=T_{2}$, the decay term goes down to 1/e. In practice, the finite pulse length leads to a deviation in the real phase term from χ(t).

Specifically, the total Hamiltonian in the rotating-wave approximation is

(35) $$H_{\delta ,\kappa }\left(t\right)=\frac{\delta +\delta_{d}\left(t\right)}{2}\sigma_{z}+g_{\mathrm{ac}}\cos\left(\omega_{s}t\right)\sigma_{z}+\kappa H_{c},$$

where a time-dependent dynamic noise term δ(t) is considered, obtained with the Ornstein–Uhlenbeck process

(36) $$\delta_{d}\left(t+\Delta t\right)=\delta_{d}\left(t\right)e^{-\Delta t/\tau }+{\left[\frac{c\tau }{2}\left(1-e^{-2\Delta t/\tau }\right)\right]}^{1/2}n,$$

where τ and *c* are the correlation time and the diffusion constant of noise, respectively, valued as τ=20μs and $\sqrt{c\tau /2}=2\pi \times 50\;\mathrm{KHz}$ [17]; and n∼N(0,1). Controlled Hamiltonian $H_{c}$ only takes a non-zero value during pulse duration $T_{\mathrm{pulse}}$. We took the frequency of the signal to be $\omega_{s}=\pi /\left(T_{\mathrm{pulse}\;}+\tau_{\mathrm{pulse}}\right)=2.5\pi \;\mathrm{MHz}$, where $\tau_{\mathrm{pulse}}$ is the time separation between two adjacent pulses. We took the maximum limit of the field amplitude as $\Omega_{\max}=2\pi \times 10\;\mathrm{MHz}$; so, for the rectangular pulse, $T_{\mathrm{pulse}\;}=50\;\mathrm{ns}$, during which $H_{c}=\Omega_{\max}\sigma_{x}$ for the Pauli-X gate and $H_{c}=\Omega_{\max}\sigma_{y}$ for the Pauli-Y gate, and the time separation was $\tau_{\mathrm{pulse}}=350\;\mathrm{ns}$. For the optimization pulses, we set $T_{\mathrm{pulse}\;}=100\;\mathrm{ns}$ and $\tau_{\mathrm{pulse}}=300\;\mathrm{ns}$.

## 3. Results

### 3.1. Feasibility of the Estimation Model

**Figure 3:**
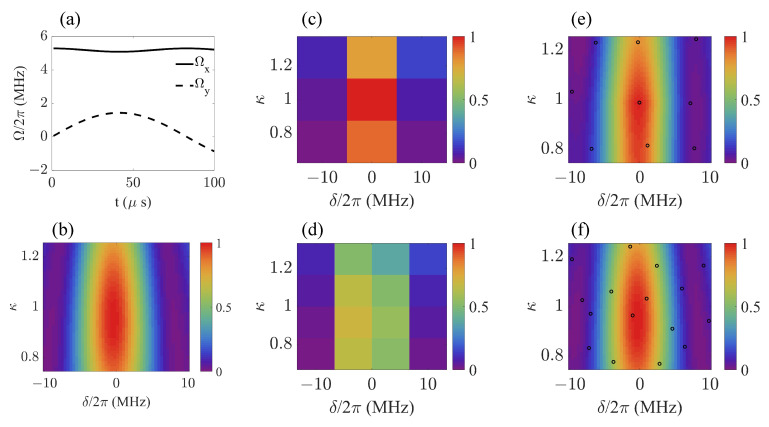
(**a**) Shape of control field in interaction picture, $\Omega_{x}^{\mathrm{PM}}\left(t\right)=\sum_{j=1}^{N_{D}}\frac{a_{j}}{2}\cos\left[\frac{b_{j}}{v_{j}}\sin\left(v_{j}t\right)\right]$ and $\Omega_{y}^{\mathrm{PM}}\left(t\right)=\sum_{j=1}^{N_{D}}\frac{a_{j}}{2}\sin\left[\frac{b_{j}}{v_{j}}\sin\left(v_{j}t\right)\right]$, with randomly taken parameters a=0.0332, b=0.0104 and ν=0.0378. (**b**,**c**) Values of true function with sample number $N_{s}=9$. (**d**) Values of true function with sample number $N_{s}=16$. (**e**) Values of estimation function with sample number n=9 and estimation point number $N_{e}=2500$. (**f**) Values of estimation function with sample number n=16 and estimation point number $N_{e}=2500$. Black circles in (**e**,**f**) represent locations of sample points.

We first demonstrate the feasibility of the estimation model based on its estimation accuracy and time efficiency. Figure 3 shows its performance in predicting the fidelity of the final state under certain frequency detuning δ and amplitude drift factor κ. For the fixed control field shown in Figure 3a, the value of f(δ,κ) varied with δ and κ, which we show with a 2D color distribution image in Figure 3b, where 50×50 sample points were taken to ensure its resolution. If the available sample number decreased to 9 and 16, respectively, the resolution ratio clearly dropped, as shown in Figure 3c,d. However, based on the same number of sample points, the images depicted with 2500 prediction values $\hat{f}\left(x\right)$ in Figure 3e,f show an obvious improvement in resolution ratio and accuracy compared with their counterparts in Figure 3c,d, respectively. By applying the estimation method, more information about how the target function distributed in the parameter space was extracted based on the knowledge provided by the sample points.

**Figure 4:**
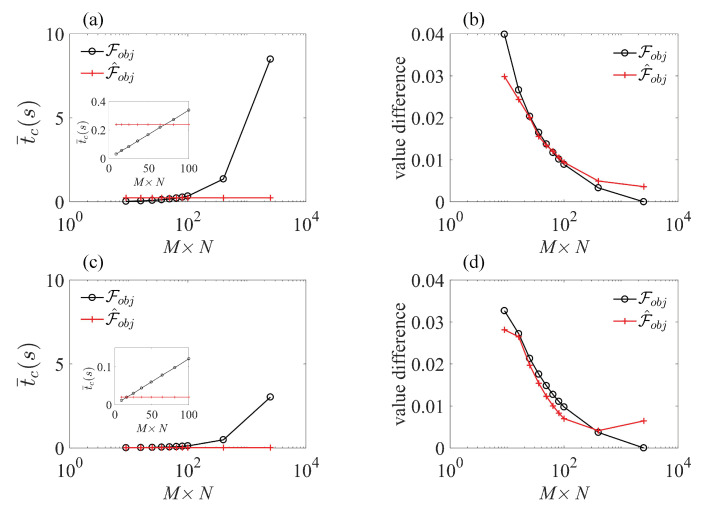
(**a**) Average computation time of the objective function as a function of calculating sample number M×N. In total, 100 processes under random control fields were calculated, and the estimation model parameter was updated in each process when computing ${\hat{F}}_{\mathrm{obj}}$. Insert: Zoomed-in graph for M×N≤100. (**b**) Average function value deviation as a function of calculating sample number M×N. Based on the same data as (**a**). The deviation was calculated by subtracting the values of f(δ,κ), with M×N=2500, i.e., $|F_{\mathrm{obj}}-F_{\mathrm{obj}(M\times N=2500)}|$ for the true value-based process and $|{\hat{F}}_{\mathrm{obj}}-F_{\mathrm{obj}(M\times N=2500)}|$ for the predictor-based process. (**c**) Average computation time of the objective function as a function of calculating sample number M×N, where the estimation model parameter was fixed in all the 100 random processes. Insert: Zoomed-in graph for M×N≤100. (**d**) Average function value deviation as a function of calculating sample number M×N. Based on the same data as (**c**). The control fields took the form of $g\left(t\right)=a\cos\left[\omega_{0}t+\frac{b}{\nu }\sin\left(\nu t\right)\right]$, with evolution time T=100 ns, and a,b and ν being randomly taken from ranges a∈[0,10×2π] MHz, b∈[0,2π/T] and ν∈[0,2π/T]. The sample number used in the estimation model was n=16.

Another concern is how much time this prediction process takes, or if it has advantage in terms of computing time and cost. Here, we used the average computing time of 100 prediction processes to give a general description. The control field in each process took the form of $g\left(t\right)=a\cos\left[\omega_{0}t+\frac{b}{\nu }\sin\left(\nu t\right)\right]$, with evolution time T=100 ns, and parameters a,b and ν were randomly taken from the ranges of a∈[0,10×2π] MHz, b∈[0,2π/T] and ν∈[0,2π/T]. Each prediction process used 16 sample points, and the mission was to calculate the average fidelity (see Equation (11)) in ranges δ∈2π×[−10,10] MHz and κ∈[0.5,1.5]. We represent the estimated fidelity as

(37) $${\hat{F}}_{\mathrm{obj}}=N\sum_{k=1}^{M}\sum_{j=1}^{N}p\left(\delta_{k}\right)p\left(\kappa_{j}\right)\hat{f}\left(\delta_{k},\kappa_{j}\right),$$

where the formation and values of $N,p\left(\delta_{k}\right)$ and $p\left(\kappa_{k}\right)$ are the same as in Equation (11), and M×N represents the number of different prediction points used in the calculation. Two strategies were separately applied: in Figure 4a, the estimation model was updated in each trial, while in Figure 4c, a fixed estimation model served in all the 100 trials. To make a comparison, we also show the results of calculating the true $F_{\mathrm{obj}}$ with M×N sample points. It is natural that for the true function-based objective function $F_{\mathrm{obj}}$, the computation time was approximately proportional to M×N, the total number of function f(δ,κ) calling times. In contrast, the processing time of the predict function-based objective function remained stable as M×N increased, causing distinct time retrenching as M×N increased to 2500. Notice here that for M×N≤64, the computation time of ${\hat{F}}_{\mathrm{obj}}$ was longer than that for $F_{\mathrm{obj}}$, because the values of 16 sample points needed to calculated, and an extra amount of time needed to be spent on selecting the parameters that constructed the estimation model. Figure 4c shows that once the estimation model was built, the process for calculating the value of the predict function became rapid, and its corresponding time consumption could be neglected, so the total computation time was decided by the number of true sample points, i.e., M×N=16.

Figure 4b,d show the estimation accuracy of ${\hat{F}}_{\mathrm{obj}}$ and $F_{\mathrm{obj}}$ with different calculated sample numbers M×N. In Figure 4b, we used the same data as in Figure 4a, and in Figure 4d, we used the same data as in Figure 4c. We used the value of $F_{\mathrm{obj}}$ with M×N=2500 as the benchmark to calibrate the accuracy, denoted as $F_{\mathrm{obj}(M\times N=2500)}$. The estimation accuracy was expressed as the value deviation from $F_{\mathrm{obj}(M\times N=2500)}$, that is, $|{\hat{F}}_{\mathrm{obj}}-F_{\mathrm{obj}(M\times N=2500)}|$ with the estimation objective function method and $|F_{\mathrm{obj}}-F_{\mathrm{obj}(M=50)}|$ with the true objective function method. In general, the deviation decreased as M×N increased, and the estimation deviation values of Figure 4b,d were in the same scope, showing that the fixed estimation model strategy did not damage the estimation accuracy.

### 3.2. Optimization Efficiency of the B-PM Method

**Figure 5:**
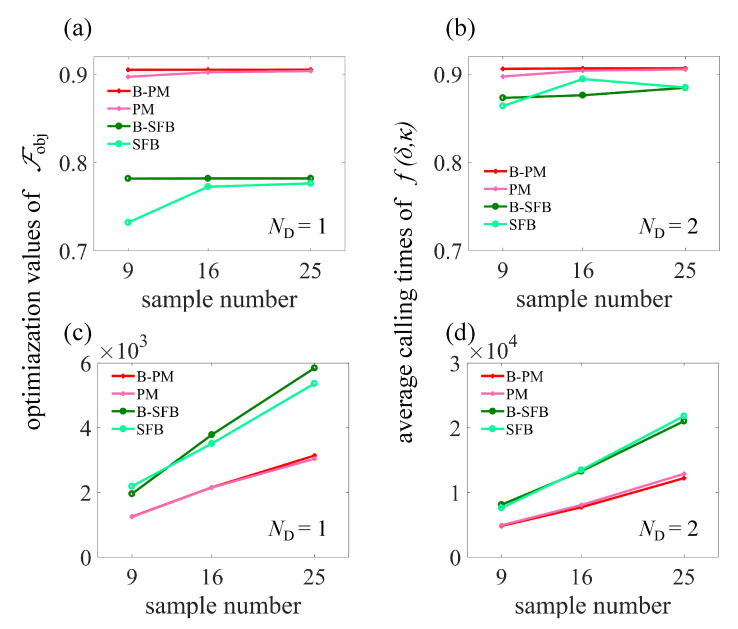
(**a**) Optimizedfidelity of B-PM, PM, B-SFB and SFB methods with parameter set number $N_{D}=1$. (**b**) Optimized fidelity of B-PM, PM, B-SFB and SFB methods with parameter set number $N_{D}=2$. (**c**,**d**) Average function f(δ,κ) calling times in Equation (6) that gave the results in (**a**,**b**), respectively. All results are based on 100 trials with random initial parameters. The total evolution time was taken as T=100 ns, and the maximal field amplitude was bounded as $\max|g\left(t\right)|⩽\Omega_{\max}=2\pi \;\times \;10$ MHz.

The results in Figure 4 demonstrate that by using the fixed estimation model, the total computation time is decided by the function f(δ,κ) calling times. Figure 5 shows a comparison among the B-PM method, the PM method, the Bayesian estimation SFB (B-SFB) method and the SFB method in terms of the final value of $F_{\mathrm{obj}\;}$ and the calling times of function f(δ,κ) during the search process. The results with $N_{D}=1$ are shown in Figure 5a,c, and the results with $N_{D}=2$ are shown in Figure 5b,d. Each result is based on 100 trials with random initial parameters. On the whole, the B-PM method gave the result of $F_{\mathrm{obj}}=0.905$ with 1252 average calling times, while the SFB method gave the result of $F_{\mathrm{obj}}=0.894$ with 13,479 average calling times, so B-PM resulted in improved average fidelity using only 9.3% of computation resources compared with SFB method.

**Figure 6:**
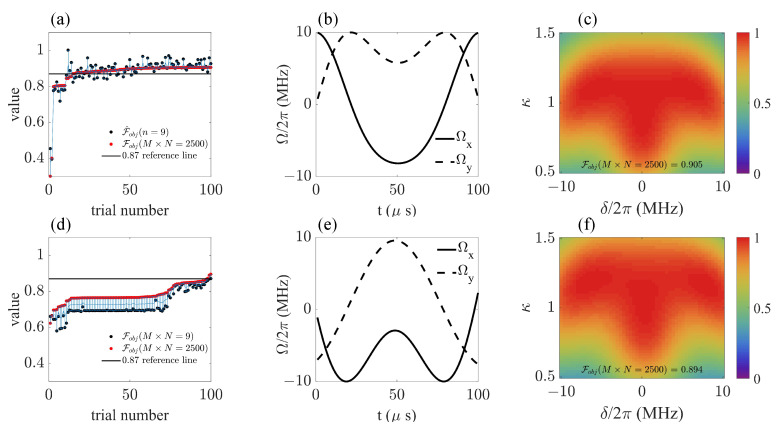
(**a**–**c**) Optimization results obtained with the B-PM method with $N_{D}=1$ and n=9. (**d**–**f**) Optimization results obtained with the SFB method with $N_{D}=2$ and M×N=16. (**a**,**d**) Optimization values of objective function of 100 trials with random initial parameters λ. (**b**,**e**) Shape of the optimized control field in the interaction picture. (**c**,**f**) Fidelity distribution in regions δ∈2π×[−10,10] MHz and κ∈[0.5,1.5].

Figure 6 shows the detailed optimization results of the B-PM method with $N_{D}=1$ and n=9 and those of the SFB method with $N_{D}=2$ and M×N=16. Figure 6a,d show the final objective function values of 100 trials with random initial parameters λ, in which we marked the value used in the optimization process with black points and their corresponding, more accurate values calculated with M×N=2500 true sample points with red points. As is shown in Figure 6a, among 100 trials of the B-PM method, 42 results gave $F_{\mathrm{obj}}\geq 0.9$, and 86 results gave $F_{\mathrm{obj}}\geq 0.87$, while with the SFB method (Figure 6b), only 3 results out of 100 trials gave $F_{\mathrm{obj}}\geq 0.87$. Figure 6b,e display the shape of the optimized control field in the interaction picture, and Figure 6c,f display the fidelity distribution in regions δ∈2π×[−10,10] MHz and κ∈[0.5,1.5].

### 3.3. Sensitivity Improvement in AC Magnetometry

**Figure 7:**
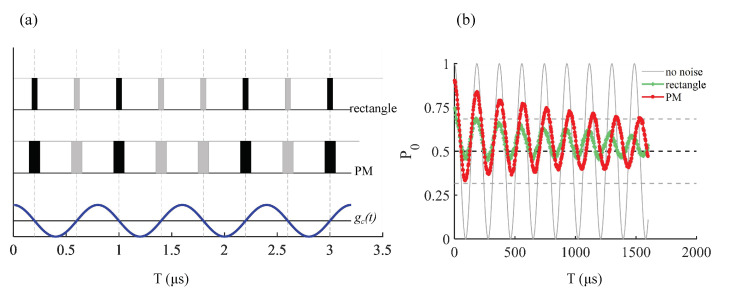
(**a**) Scheme of XY-8 pulse and AC signal to be sensed. The pulse length of the rectangular pulse was $T_{\mathrm{pulse}}=50$ ns, and that of the PM pulse was $T_{\mathrm{pulse}}=100$ ns. The time separation for the rectangular pulse was τ=350 ns, and that for the PM pulse was τ=300 ns. The frequency of the AC signal was $\omega_{s}=\pi /\left(T_{\mathrm{pulse}}+\tau \right)=2.5\pi$ MHz. (**b**) Simulation results of the population of |0〉 of the NV center ensemble using different XY-8 pulses. Red line with point marks: population under optimized pulse obtained with the B-PM method with pulse length $T_{\mathrm{pulse}}=100$ ns. Green line with cross marks: population under rectangular π pulse with pulse length $T_{\mathrm{pulse}}=50$ ns. Gray solid curve: population under rectangular π pulse with pulse length $T_{\mathrm{pulse}}=100$ ns without considering inhomogeneous broadening or the dynamic noise term. Gray dashed lines: from top to bottom, $P_{0}=\left(1+1/e\right)/2$ reference, $P_{0}=1/2$ reference and $P_{0}=\left(1-1/e\right)/2$ reference, respectively. The $T_{2}$ time was identified as the time when the maximal value of $P_{0}$ dropped below (1+1/e)/2.

Figure 7a shows the sketch of the XY-8 sequence and AC signal to be sensed. Figure 7b shows the population of the sensor in |0〉, measured at every terminal of a single XY-8 period so that the time interval between two data points was 3.2μs. The 1000 measurements made under different δ obeyed the Gaussian distribution with zero mean value and FWHM = 2π×26.5MHz, and the $T_{2}$ time was identified as the time when the maximal value of $P_{0}$ dropped below (1+1/e)/2, the value of which is marked with a reference gray dashed line in Figure 7b. Because of the inhomogeneous broadening of δ as well as dynamic noise δ(t), the populations decayed over time, i.e., $T_{2}\approx 180\;\mu s$ for the rectangular XY-8 pulse and $T_{2}\approx 1500\;\mu s$ for the PM XY-8 pulse, respectively.

## 4. Discussion

Our work shows that the Bayesian-based estimation model can be effectively applied to estimating the fidelity of the state transformation and the gate construction of NV centers under wide inhomogeneous broadening noise. The estimation accuracy as well as the time efficiency make it an ideal tool to constitute a new practical optimization method, which we denote as Bayesian estimation phase-modulated (B-PM) method. The B-PM method can be combined with various of search algorithms, i.e., the direct search algorithm [43,44], the genetic algorithm [45] and the gradient-based algorithm [46,47,48,49]. Here, we adopted the widely used Nelder–Mead direct search method [50] and related optimization tools that are easily available on the most common programming platforms. The Nelder–Mead method seeks the best parameter point by successively constructing new points to replace the worst point following a heuristic simplex approach. Consequently, the objective function needs to be frequently called to rank the points during the searching process, which consumes the majority of the total processing time. The hybrid Bayesian estimation phase-modulated (B-PM) method reduces the processing time in two ways. On the one hand, by setting the predict function as the objective function, the time needed to compute the objective function value once is reduced, as shown in Figure 4. On the other hand, the phase-modulated method can significantly decrease the total number of times in which objective functions are called, by introducing a simpler landscape of the parameter space. This is based on its ability to comprise more frequencies with the same number of parameters than the conventional SFB and other amplitude-modulated methods. A detailed description about this point can be found in reference [29], and recent works [51] using other kinds of phase-modulated control methods also show their usability in protecting the coherence of samples with large inhomogenities.

Taking these two factors together, the advantage of the B-PM method over the commonly used standard Fourier basis(SFB) method becomes obvious. To be specific, compared with the best result obtained with the SFB method, the B-PM method increased the optimal value of the objective function from 0.894 to 0.905, with a more than 90% decrease in the average time consumption. Moreover, among 100 results obtained with random initial parameters, only 1 result of the SFB ($N_{D}=2$ and M×N=16) method gave $F_{\mathrm{obj}}\geq 0.89$, while 42 results of the B-PM ($N_{D}=1$ and n=9) method gave $F_{\mathrm{obj}}\geq 0.9$, indicating that the B-PM method is much more robust and can reach a fine result when the amount of total trials is limited. When making a comparison between the B-PM and PM methods, people may query that the improvement induced by the Bayesian estimation model is not obvious. This is true for the current system studied in this article, which needs to be tested on more physical models and systems. However, we stress here that based on the results showed in Figure 5, the B-PM method guarantees a higher value of objective function than the PM method when the computation resources are on the same scale. This is crucial when the computation resources are severely stressed and when the control performance is sensitive to the precise value of the objective function.

We further displayed the utility of the B-PM method in NV center-based AC magnetometry, especially in high-frequency cases where adiabatic strategies are not viable. The numerical simulation result shows that the optimized pulse achieved an eight-fold extension of coherence time $T_{2}$ compared with the conventional rectangular π pulses with the same maximum amplitude. A similar optimization strategy can applied to other sensing cases, e.g., the spin bath driving [22,52,53] process for DC magnetometry sensing. We used the XY8 sequence in our simulation, but the optimization pulses can be applied to other DD sequences, including XY16 [54], periodic dynamical decoupling (PDD) [55], concatenate dynamical decoupling (CDD) [56] and so on [57]. Comparing the performance of optimization pulses in these sequences could provide a useful prescription for DD optimization, which will be completed in our subsequent studies. We denote that the normal distribution in Equation (3) results from a variety of disturbance factors, including the interaction between NV center spins, nearby nuclear spins or spin bath, and external bias fields, as well as the frequency bias of the control field. We took the FWHM of the broadening as 2π×26.5 MHz in our simulation, corresponding to a dephasing time of $T_{2}^{*}\approx 20$ ns. To check the feasibility of the B-PM method under other conditions with longer $T_{2}^{*}$, we simulated the fidelity of the Pauli-X gate for $T_{2}^{*}\approx 20$ ns, $T_{2}^{*}\approx 260$ ns (corresponding FWHM =2π×2 MHz) and $T_{2}^{*}\approx 2600$ ns (corresponding FWHM =2π×0.2 MHz). The B-PM control field gave the results of 0.8454, 0.9671 and 0.9804, respectively, while the rectangular pulse gave the results of 0.8047, 0.9583 and 0.9628. These results show that our method is feasible under different $T_{2}^{*}$ conditions.

One important potential application area of the B-PM method is the closed-loop optimization [25,26,27] of complex systems, such as many-body [58] and many-electron systems [59]. In these cases, numerical simulations fail to accurately describe the system due to a lack of information of the complex system, and the experimental results are directly used as the values of the objective function in the optimization process. The advantage of the B-PM method in reducing the requisite total sample number during the process could be more prominent in such conditions, since the time needed to experimentally obtain one sample dataset is generally much longer than that of the simulation process. Besides NV centers, the B-PM method is also expected be applied to the optimization of other prevailing quantum platforms, such as trapped ions [60,61,62], cold atoms and superconducting qubits [63,64].

## Data Availability

Data are available from the corresponding author.

## Abbreviations

The following abbreviations are used in this manuscript:

PM	Phase-modulated
B-PM	Bayesian estimation phase-modulated
SFB	Standard Fourier basis
B-SFB	Bayesian estimation standard Fourier basis
FWHM	Full width at half maximum
DD	Dynamical decoupling
AC	Alternating current
DC	Direct current
PDD	Periodic dynamical decoupling
CDD	Concatenate dynamical decoupling
